# Correction: Horne et al. White Matter Correlates of Domain-Specific Working Memory. *Brain Sci*. 2023, *13*, 19

**DOI:** 10.3390/brainsci14050475

**Published:** 2024-05-08

**Authors:** Autumn Horne, Junhua Ding, Tatiana T. Schnur, Randi C. Martin

**Affiliations:** 1 Department of Psychological Sciences, Rice University, Houston, TX 77005, USA; 2 Department of Neurosurgery and Neuroscience, Baylor College of Medicine, Houston, TX 77030, USA; jhding@mail.bnu.edu.cn (J.D.);; 3 Department of Psychology, University of Edinburgh, Edinburgh EH8 9YL, UK

## Error in Figure/Table

In the original publication [1], there were mistakes in Table 3 as published. There were errors in the dataset that was the basis of the analyses for this paper for eight out of forty-five participants. The corrected Table 3 appears below.

In the original publication, there were mistakes in Table 4 as published. There were errors in the dataset that was the basis of the analyses for this paper for eight out of forty-five participants. The corrected Table 4 appears below.

In the original publication, there were mistakes in Table 5 as published. There were errors in the dataset that was the basis of the analyses for this paper for eight out of forty-five participants. The corrected Table 5 appears below.

In the original publication, there were mistakes in Table 6 as published. There were errors in the dataset that was the basis of the analyses for this paper for eight out of forty-five participants. The corrected Table 6 appears below.

In the original publication, there were mistakes in Table 7 as published. There were errors in the dataset that was the basis of the analyses for this paper for eight out of forty-five participants. The corrected Table 7 appears below.

In the original publication, there were mistakes in Table 8 as published. There were errors in the dataset that was the basis of the analyses for this paper for eight out of forty-five participants. The corrected Table 8 appears below.

In the original publication, there were mistakes in Table 9 as published. There were errors in the dataset that was the basis of the analyses for this paper for eight out of forty-five participants. The corrected Table 9 appears below.

In the original publication, there were mistakes in Figure A2 as published. There were errors in the dataset that was the basis of the analyses for this paper for eight out of forty-five participants. The corrected Figure A2 appears below.

## Missing Citation

In the original publication, Miller, E.K.; Buschman, T.J. Working memory capacity: Limits on the bandwidth of cognition. *Daedalus* **2015**, *144*, 112–122. https://doi.org/10.1162/DAED_a_00320 was not cited. The citation has now been inserted in *Section 1.3. White Matter Correlates of Domain-Specific WM*, Paragraph 1 and should read: Miller and Buschman (2015) [31] applied this idea in the WM domain by proposing that, if cognitive functions, such as WM, rely on the synchronous activity of a brain network, then a greater range of possible neuronal oscillation frequencies would facilitate synchronous activity between the regions involved in WM processes.

In the original publication, Benjamini, Y.; Hochberg, Y. Controlling the False Discovery Rate: A Practical and Powerful Approach to Multiple Testing. *J. R. Stat. Soc. Ser. B (Methodol.)* **1995**, *57*, 289–300 was not cited. The citation has now been inserted in *Section 3.2. Tract Integrity and WM*, Paragraph 1 and should read: Using the FDR correction for multiple comparisons (Benjamini and Hochberg, 1995) [60], separately, for the pairwise relations to semantic and phonological WM, phonological WM was related to the whole AF, the direct segment of the AF, the posterior segment of the AF, and the ILF.

## Text Correction

There were errors in the original publication. There were errors in the dataset that was the basis of the analyses for this paper for eight out of forty-five participants.

A correction has been made to the **Abstract**.

Prior evidence suggests domain-specific working memory (WM) buffers for maintaining phonological (i.e., speech sound) and semantic (i.e., meaning) information. The phonological WM buffer’s proposed location is in the left supramarginal gyrus (SMG), whereas semantic WM has been related to the left inferior frontal gyrus (IFG), the middle frontal gyrus (MFG), and the angular gyrus (AG). However, less is known about the white matter correlates of phonological and semantic WM. We tested 45 individuals with left hemisphere brain damage on single word processing, phonological WM, and semantic WM tasks and obtained T1 and diffusion weighted neuroimaging. Virtual dissections were performed for each participants’ arcuate fasciculus (AF), inferior fronto-occipital fasciculus (IFOF), inferior longitudinal fasciculus (ILF), middle longitudinal fasciculus (MLF), and uncinate fasciculus (UF), which connect the proposed domain-specific WM buffers with perceptual or processing regions. The results showed that the left IFOF and the posterior segment of the AF were related to semantic WM performance. Phonological WM was related to both the left ILF and the whole AF. This work informs our understanding of the white matter correlates of WM, especially semantic WM, which has not previously been investigated. In addition, this work helps to adjudicate between theories of verbal WM, providing some evidence for separate pathways supporting phonological and semantic WM.

A correction has been made to **Results**, **Sections 3.2–3.7**.

## *3.2. Tract Integrity and WM* 

In all continuous multiple regression models reported here, tract FA was regressed on phonological WM (digit matching), semantic WM (category probe), phonological single-word processing (phonological d’), semantic single-word processing (semantic d’), and the cube root of gray matter damage to the tracts’ termination regions. We transformed the measures of percent damage to gray matter regions by taking the cube root because the distribution of gray matter damage was highly negatively skewed. The predicted relationships between left hemisphere tracts and phonological or semantic WM are outlined in Table 4, in terms of their independent contribution in the multiple regression. The pairwise correlations between the left hemisphere white matter tract FA values and the behavioral measures are presented in Table 5. Using the FDR correction for multiple comparisons (Benjamini and Hochberg, 1995) [60], separately, for the pairwise relations to semantic and phonological WM, phonological WM was related to the whole AF, the direct segment of the AF, the posterior segment of the AF, and the ILF. Semantic WM was related to the direct segment of the AF, the posterior segment of the AF, the IFOF, and the ILF. However, although the pairwise results suggested several relations between tract FA and both phonological and semantic WM, it is important to factor in single-word processing and gray matter damage to terminations because, for example, variations in phonological processing may have reduced pairwise correlations to phonological WM, whereas variations in semantic processing may have contributed to positive correlations. The results of the continuous multiple regression analyses that tested the hypothesized relations between left hemisphere tracts and WM, while including all the control variables, are presented in Table 6. As shown there, two tracts showed significant weights for semantic WM and two for phonological WM. Phonological WM was related to the integrity of the whole AF and the ILF. Semantic WM, on the other hand, was related to the posterior portion of the AF and the IFOF. In regard to correcting for multiple comparisons, the FDR correction cannot be directly applied to the results from several multiple regression analyses. We note, however, that if we treated the 16 total weights for semantic and phonological WM as independent observations, one might have expected that less than one weight would have been significant by chance alone (0.05 × 16 = 0.8) for alpha = 0.05. Thus, the fact that four weights were significant, greatly exceeds this number and strongly suggests that most relations observed here were not due to chance. Additional analyses using logistic regression for the tracts with more than 10 untraceable tracts are presented in the following sections. For all tables, statistical results with *p* < 0.05 are presented in bold.

## *3.3. Arcuate Fasciculus (AF)* 

Our first prediction was that left AF integrity would be related to phonological WM performance. When we predicted the integrity of the whole left AF FAs using continuous regression, the weight for phonological WM was significant but semantic WM was not. Further, when we predicted the integrity of the posterior subsection of the AF, the weight for semantic WM was significant but phonological WM was not (Table 6). Because there were many untraceable tracts for the AF and its subsections (Table 2) and because prior studies had implicated the AF in phonological WM (e.g., Takeuchi et al., 2011 [35]; Charlton et al., 2010 [39]), we also utilized logistic regression to test the relation between the AF and phonological and semantic WM. We predicted the presence of the AF subsections which have terminations in the SMG, the anterior, and the posterior AF would be related to phonological WM. However, the logistic regression results did not support this prediction (Table 7). Because the direct segment connects temporal lobe semantic regions to frontal regions, we also predicted that semantic WM would be related to the integrity of the direct segment of the AF, but again, the logistic regression results did not support this prediction (Table 7).

## *3.4. Inferior Fronto-Occipital Fasciculus (IFOF)* 

As predicted, the weight for semantic WM but not phonological WM was significant in the continuous multiple regression model predicting the left IFOF (Table 6). The logistic regression results mirrored the results of the continuous regression in that semantic but not phonological WM predicted the presence of the left IFOF (Table 8).

## *3.5. Inferior Longitudinal Fasciculus (ILF)* 

When we predicted left ILF FA values, the weight for phonological but not semantic WM was significant (Table 6).

## *3.6. Middle Longitudinal Fasciculus (MLF)* 

In the model predicting the left MLF FA, neither the weight for phonological nor semantic WM was significant (Table 6).

## *3.7. Uncinate Fasciculus (UF)* 

We did not observe a significant weight for either WM measure in the multiple regression models predicting the FA for the left UF (Table 6). Because there were many instances where the left UF could not be tracked, we also tested the relation between left UF integrity and WM using logistic regression. We predicted the presence of the UF with both WM measures, single-word processing, and the cube root of damage to UF terminations. Neither semantic nor phonological WM were significant predictors of the UF’s presence (Table 9).

[Removed final paragraph of 3.7]

There were errors in the original publication. There were errors in the dataset that was the basis of the analyses for this paper for eight out of forty-five participants.

A correction has been made to **Discussion**.

Here, we have reported the relationships between white matter tract integrity and domain-specific WM in a large (N = 45) group of people with left hemisphere brain damage. We predicted that phonological WM would be related to the integrity of the left AF’s anterior and posterior segments. Additionally, we predicted that semantic WM would be related to the integrity of the left direct segment of the AF, IFOF, ILF, MLF, and UF. Our predictions regarding the white matter correlates of phonological and semantic WM were based on the terminations of these tracts. Thus, we predicted that a tract would be involved in phonological WM if it terminated in the SMG and semantic WM if it terminated in the IFG or AG. A summary of the predicted and observed relations between left hemisphere tracts and WM performance is presented in Table 4.

Our predictions for the white matter correlates of phonological WM were partially supported. We reported a relation between the integrity of the whole AF and phonological WM, replicating past work reporting relationships between measures of frontoparietal tract integrity and phonological WM performance [9–11]. In addition to the relation between the AF and phonological WM, we also observed an unpredicted relationship between phonological WM and the left ILF. While the ILF connects the temporal lobe with the inferior parietal and occipital lobes, we do not have a detailed understanding of where exactly this tract terminates. While there are certainly distinct patterns, there is also an amount of observed heterogeneity, particularly in brains that have been altered because of brain damage. While we assume the ILF is more often associated with the AG, a semantic WM buffer, it is possible that it has some terminations in the nearby SMG, the proposed phonological WM buffer as well.

Our predictions for the white matter correlates of semantic WM were also partially supported. The relationship between semantic WM and the IFOF came out as expected. The left IFOF has terminations in frontal regions including the left IFG, which is a proposed semantic WM buffer region [5,6]. We propose that the left IFOF connects gray matter regions in the temporal lobes supporting semantic processing with the IFG, allowing for information in perceptual and semantic processing regions to be transferred to the IFG for semantic maintenance. There is also evidence that, in some people, the IFOF includes terminations in the precuneus region, which includes the AG [61]. Thus, another explanation for the IFOF’s relation to semantic WM could be that it connects two semantic WM regions, the IFG and the AG, as part of a larger network supporting semantic WM. Unexpectedly, we also observed a relationship between the posterior segment of the AF and semantic WM. As with the ILF, we observed heterogeneity in where exactly the posterior segment of the AF terminated in the parietal lobe. Considering the proximity of the SMG (proposed phonological WM buffer) and the AG (proposed semantic WM buffer), it is entirely possible that our method of segmenting each individual patient’s tract in their native space meant that the posterior AF was, in at least a subset of our patients, connecting the left AG with the temporal lobe.

We did not observe support for the relationships we predicted between semantic WM and the left direct AF, ILF, MLF, or UF in the multiple regression analyses. The direct segment of the AF also has terminations in perceptual processing regions in the temporal lobe, that we predicted would allow it to transfer semantic information from processing regions to the IFG for storage. Similarly, the left ILF and MLF were predicted to support semantic WM because they have terminations in the occipital and inferior parietal lobe, which includes the AG region, as well as the anterior temporal region. In both cases, we predicted that the white matter tracts allow for the semantic knowledge stored in anterior temporal regions to pass to the AG, a semantic WM buffer [56]. What are some possible explanations for why many of the semantic WM predictions were not supported? For the direct AF, ILF, and MLF, it may be that the region of the temporal lobe that these tracts terminate in is not critical for semantic processing across modalities. The hub-and-spoke-model of semantic processing proposes a modality-invariant hub coordinating semantic information across the distributed semantic processing regions [62]. Originally, it was proposed that this hub was located in the anterior temporal lobe [62]. However, more recent evidence has suggested there are gradations within the ATL where modality-invariant semantic processing is related to more middle and inferior portions of the temporal lobe, including the anterior fusiform gyrus [63]. Thus, it may be that while the ILF, MLF, and direct AF all have terminations in the temporal lobe, these terminations may not be in regions supporting modality-invariant processing, which would be most critical for semantic WM. Finally, while we predicted that the UF would be related to semantic WM because it provides a direct connection between the IFG and the anterior temporal lobe, we did not observe a relationship between the UF and WM after accounting for the contribution of other effects using our multiple regression approach. However, while the UF terminates in orbital frontal regions that include an area implicated in aspects of semantic processing (i.e., Brodmann’s area 47; Poldrack et al., 1999) [64], prior studies specific to WM for semantic information have revealed more posterior IFG regions (e.g., Hamilton et al., 2009 [5]).

Our findings about the neural correlates of WM also contribute to our theoretical understanding of WM. Specifically, understanding the neural basis of WM delineates between two buffer models of WM: the multicomponent model of WM and the domain- specific model of WM. The multicomponent model of WM includes a phonological loop which maintains phonological information and an episodic buffer which integrates (and supports the maintenance of) phonological, semantic, and visuospatial information. In contrast, the domain-specific model of WM contains separable buffers for phonological and semantic WM. While the domain-specific WM predicts distinct white matter correlates of phonological and semantic WM, the multicomponent model of WM does not. We did not observe any overlap between the tracts supporting phonological versus semantic WM in our multiple regression analyses. The multicomponent model cannot account for tracts that are only related to semantic WM performance after controlling for phonological WM performance and vice versa. While the domain-specific model of WM contains a buffer specific to semantic WM, the multicomponent model of WM does not. The episodic buffer in the multicomponent model is conceptualized as a capacity for combining phonological, semantic, and visual representations into a cohesive episodic memory. We would expect that if the tracts related to only semantic WM were the neural basis of the episodic buffer, then they should have an independent relation to phonological WM performance as well. Thus, the evidence of neural correlates distinct to semantic WM or phonological WM is most closely aligned with the domain-specific model of WM.

While this work does address many of the limitations of past work on the white matter correlates of WM, it does have its own unique set of limitations that should be addressed in future work. First, a strength of this work was its large sample size, especially for a neuropsychological investigation, but the sample size was achieved by (1) combining neuroimaging and behavioral data from participants recruited from one institution over the course of 15 years and several updates in scanning technology and protocol and (2) adding to that data collected at a different institution and scanning facility. While some past work has suggested that it is feasible to combine diffusion-weighted data collected across multiple institutions in the analyses [48], more recent work has called that claim into question and suggested ways to mitigate the effects of including data collected via different scanners and/or with different scanning protocols [59]. We would note, however, that when a scanner site is simply included as a covariate in the continuous regression models tested here, all of the previously reported significant effects remain significant (*p* = 0.0081–0.035).

## References Section Changes

The following references have been added to the Reference section:

31. Miller, E.K.; Buschman, T.J. Working memory capacity: Limits on the bandwidth of cognition. *Daedalus* **2015**, *144*, 112–122. https://doi.org/10.1162/DAED_a_00320.
60. Benjamini, Y.; Hochberg, Y. Controlling the False Discovery Rate: A Practical and Powerful Approach to Multiple Testing. *J. R. Stat. Soc. Ser. B (Methodol.)* **1995**, *57*, 289–300.
61. Vassal, F.; Schneider, F.; Boutet, C.; Jean, B.; Sontheimer, A.; Lemaire, J.-J. Combined DTI Tractography and Functional MRI Study of the Language Connectome in Healthy Volunteers: Extensive Mapping of White Matter Fascicles and Cortical Activations. *PLoS ONE* **2016**, *11*, e0152614.
62. Jefferies, E.; Lambon Ralph, M.A. Semantic impairment in stroke aphasia versus semantic dementia: A case-series comparison. *Brain* **2006**, *129*, 2132–2147.
63. Lambon-Ralph, M.A.; Jefferies, E.; Patterson, K.; Rogers, T.T. The neural and computational bases of semantic cognition. *Nat. Rev. Neurosci.* **2017**, *18*, 42–55.

With this correction, the order of some references has been adjusted accordingly. The authors state that the scientific conclusions are unaffected. This correction was approved by the Academic Editor. The original publication has also been updated.

## Figures and Tables

**Figure A2:**
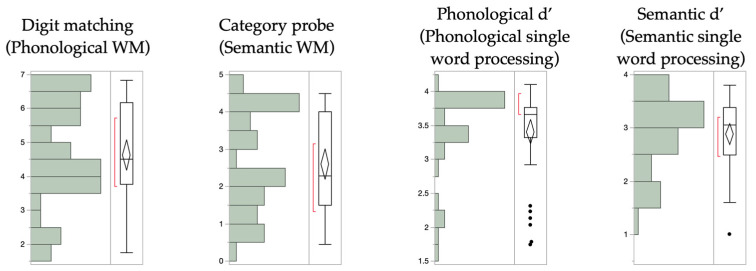
Distributions for all WM and single-word processing measures.

**Table 3:** Descriptive statistics for WM and the single word processing measures.

Measure	N	Mean	SD	Min	Max
Digit matching (phonological WM)	43	4.6	1.47	1.76	6.83
Category probe (semantic WM)	43	2.6	1.34	0.45	4.5
Phonological d’ (phonological single word processing)	45	3.4	0.60	1.74	4.11
Semantic d’ (semantic single word processing)	45	2.9	0.66	1.00	3.80

**Table 4:** Predicted and observed relationships between the left hemisphere tracts and WM.

	Phonological WM	Semantic WM
Left AF	●✓	
Left anterior AF	●	
Left direct AF		●
Left posterior AF	●	✓
Left IFOF		●✓
Left ILF	✓	●
Left MLF		●
Left UF		●

● = Predicted; ✓ = Observed.

**Table 5:** Pairwise correlations between the left hemisphere white matter tract FAs and the behavioral measures.

Tract	Phonological WM	Semantic WM	Phonological d’	Semantic d’
Left AF n = 27	***r* = 0.49** ***p* = 0.011**	*r* = 0.37 *p* = 0.065	*r* = −0.005 *p* = 0.98	*r* = −0.25 *p* = 0.21
Left anterior AF n = 24	*r* = 0.31 *p* = 0.16	*r =* 0.34 *p* = 0.10	*r =* −0.15 *p* = 0.49	*r =* −0.25 *p* = 0.24
Left direct AF n = 22	***r* = 0.51** ***p* = 0.014**	***r =* 0.51** ***p* = 0.019**	*r =* 0.17 *p* = 0.46	*r =* −0.25 *p* = 0.25
Left posterior AF n = 18	***r* = 0.57** ***p* = 0.014**	***r =* 0.82** ***p* = 0.0001**	*r =* −0.05 *p* = 0.85	*r =* −0.30 *p* = 0.23
Left IFOF n = 32	***r* = 0.37** ***p* = 0.043**	***r* = 0.55** ***p* = 0.001**	*r* = −0.12 *p* = 0.51	*r* = −0.15 *p* = 0.40
Left ILF n = 45	***r* = 0.49** ***p* = 0.0010**	***r* = 0.48** ***p* = 0.001**	*r* = −0.11 *p* = 0.47	*r* = 0.10 *p* = 0.52
Left MLF n = 37	*r* = 0.15 *p* = 0.38	*r* = 0.15 *p* = 0.39	*r* = −0.12 *p* = 0.47	***r* = −0.33** ***p* = 0.046**
Left UF n = 34	*r* = 0.27 *p* = 0.13	*r* = 0.26 *p* = 0.14	*r* = −0.29 *p* = 0.10	*r* = −0.050 *p* = 0.78

**Table 6:** Results of the continuous multiple regressions predicting the left hemisphere tract FA.

	Phon WM	Sem WM	Phon d’	Sem d’	Gray Matt.
**Left AF**					
Estimate	**0.012**	0.003	−0.005	−0.025	0.012
*t*	**2.41**	0.52	−0.49	−2.01	0.39
*p*	**0.026**	0.61	0.63	0.058	0.70
**Left anterior AF**					
Estimate	0.005	0.008	−0.011	−0.016	0.034
*t*	1.03	1.40	−1.15	−1.28	1.00
*p*	0.32	0.18	0.27	0.22	0.33
**Left posterior AF**					
Estimate	0.004	**0.022**	0.003	−0.009	**−0.20**
*t*	0.80	**4.06**	0.28	−0.95	**−3.00**
*p*	0.44	**0.002**	0.78	0.36	**0.011**
**Left direct AF**					
Estimate	0.013	0.004	0.007	−0.031	−0.030
*t*	1.55	0.43	0.43	−1.64	−0.63
*p*	0.14	0.67	0.68	0.12	0.54
**Left IFOF**					
Estimate	−0.002	**0.023**	−0.013	−0.015	0.010
*t*	−0.32	**2.86**	−1.14	−1.24	0.33
*p*	0.75	**0.009**	0.27	0.228	0.74
**Left ILF**					
Estimate	**0.012**	0.006	−0.021	−0.013	−0.067
*t*	**2.41**	0.93	−1.92	−1.21	−2.69
*p*	**0.021**	0.36	0.063	0.23	0.011
**Left MLF**					
Estimate	0.006	0.003	−0.002	−0.027	0.013
*t*	1.27	0.67	−0.17	−2.63	0.67
*p*	0.22	0.51	0.87	0.014	0.51
**Left UF**					
Estimate	0.007	−0.0001	−0.013	−0.007	−0.043
*t*	1.24	−0.02	−0.95	−0.53	−0.83
*p*	0.23	0.99	0.35	0.60	0.42

**Table 7:** Logistic regression models predicting the left AF and its subsections.

	Phon WM	Sem WM	Phon d’	Sem d’	Gray Matt.
**Left AF**					
Estimate	0.12	1.07	**−3.03**	0.18	**−8.45**
*χ^2^*	0.14	2.24	**4.56**	0.02	**8.11**
*p*	0.71	0.14	**0.033**	0.88	**0.004**
**Left anterior AF**					
Estimate	−0.26	0.65	**−2.68**	1.99	**−7.42**
*χ^2^*	0.29	1.41	**4.42**	2.33	**6.57**
*p*	0.59	0.24	**0.036**	0.13	**0.010**
**Left posterior AF**					
Estimate	1.11	0.15	0.33	−0.85	−13.83
*χ^2^*	2.8	0.07	0.02	0.43	2.51
*p*	0.094	0.79	0.9	0.51	0.11
**Left direct AF**					
Estimate	0.53	0.84	−1.77	−0.89	**−7.88**
*χ^2^*	1.59	2.54	3.02	0.75	**6.87**
*p*	0.21	0.11	0.082	0.39	**0.009**

**Table 8:** Logistic regression model predicting the presence of the left IFOF.

	Phon WM	Sem WM	Phon d’	Sem d’	Gray Matt.
Estimate	0.011	**1.27**	−0.29	0.031	0.37
*χ^2^*	0.0	**5.45**	0.14	0.0	0.04
*p*	0.98	**0.02**	0.71	0.97	0.83

**Table 9:** Logistic regression model predicting the presence of the left UF.

	Phon WM	Sem WM	Phon d’	Sem d’	Gray Matt.
Estimate	−0.43	1.37	2.34	−0.30	**−15.98**
*χ^2^*	0.39	2.25	2.27	0.07	**6.06**
*p*	0.53	0.13	0.13	0.80	**0.014**
